# Telemedicine: Issues in the Analysis of Its Use in Elderly People and in People with Disabilities, According to the Perspective of the Clinical Psychology of Disability

**DOI:** 10.3390/geriatrics8010005

**Published:** 2022-12-30

**Authors:** Donatella Rita Petretto, Luca Gaviano, Gian Pietro Carrogu, Roberta Berti, Martina Pinna, Roberto Pili

**Affiliations:** 1 Department of Education, Philosophy and Psychology, University of Cagliari, 09124 Cagliari, Italy; 2 IERFOP Onlus, Via Platone 1/3, 09100 Cagliari, Italy

The recent COVID-19 pandemic has led to a sudden increase in the speed of the digitization process, which has affected several areas of life (public administration, schools, universities, and healthcare, and extending to so-called “digital citizenship”). The digitization processes that spread during the COVID-19 pandemic have been defined as “emergency processes” as they were created to meet the time-sensitive need for communication, learning, and care in the most acute phases of the pandemic [1,2,3,4]. On the one hand, this sudden acceleration has not always made it possible to consider every factor necessary for the diffusion of digitization (for example, the training of operators and other people involved, reflection on infrastructural aspects and technological resources, and reflection on how to guarantee equal access opportunities for people with disabilities). On the other hand, it has enabled some processes to be activated, and allows us to reflect on the strengths and possible critical points of some experiences [1,2,3,4]_._

The acceleration of telemedicine diffusion has also occurred in the health field, a process that had already begun to consolidate over time. The pandemic emergency has enabled the activation of some processes and the consolidation of some experiences. In this article, we will specifically reflect on experiences of using the tools and methods of telemedicine in people with disabilities and in elderly people. We deem it appropriate to address this area while considering that this can be synthetically described by the so-called “digital paradox”. Regarding the digital paradox and the paradox of telemedicine, advances in this field represent a good opportunity, in a healthcare context, to enable the use of services/processes/products and information by people with disabilities (as well as elderly people in fragile condition, people in advanced stages of life, and people who reside far from healthcare services). However, if we do not adequately consider all the elements and variables that can influence the use of and access to these processes, products and telemedicine environments, it is precisely the people who would benefit most from these services who run the greatest risk of exclusion.

In this editorial, we will therefore address these issues, reflecting on some questions about equal access and inclusion opportunities and using key words that pertain to these issues. We will begin with some general definitions, which will serve as a general background.

The first group of keywords concerns the health sector and terms used in the field of telemedicine, including the concepts of telehealth and e-health.

The second group of keywords concerns specific constructs in the field of disability and the concept of disability itself. We will provide insight into the concepts of assistive technologies, reasonable accommodation, and Design for All, with a focus on the two developmental trajectories of “disability with aging” (people who encounter disabling conditions in the most advanced stages of life due to pathologies linked to age) and “aging with disability” (which refers to people who became aware of their disabling conditions in the initial stages of life, and are now able to live longer due to an increase in life expectancy) [5].

We will then reflect on a third group of terms, which concerns the digital knowledge and/or skills necessary to use the tools and methods of telemedicine, as well as the infrastructure (availability of the network connection) and the necessary devices (computers, tablets, smartphones, and other specific tools).

The definition of telemedicine has developed over time from its first reference in 1970 [6,7] to the proposal made by the World Health Organization (WHO). In the late 1990s [8], WHO defined telemedicine as a “provision of health services in which distance is a critical factor, in which all health professionals use digital technologies for the exchange of valid information for the diagnosis, treatment and prevention of disorders and damages, for research, evaluation and for the development of continuing education of health professionals, all with the aim of promoting the advancement of individual and community health” [8]. More recently, telemedicine has been described as “that branch of medicine that makes use of technological tools for the diagnosis and treatment of patients remotely”. Although it is sometimes considered a sort of synonym, the term telehealth refers to a specific area of telemedicine, relating to a set of services that can provide remote healthcare. E-health, on the other hand, refers to the broader concept of the general use of technologies in a healthcare context, even when a clinical professional figure is not necessarily present. Since the 1990s, this term has been used to describe the use of technologies and the Internet to improve or allow access to knowledge and services in the health sector. Today this concept has been further extended to include the experiences of all the “actors” involved, as well as all the services/products/processes/infrastructures related to digitization in the health sector. Therefore, in this work, we will also focus on equal access opportunities for people with disabilities and for elderly people to both the tools and processes of telemedicine, but also to the more general field of e-health.

Regarding the second group of keywords, these relate to reflection on the current concept of disability, a concept that has taken on different meanings, both over time and through different approaches and conceptual models. Currently the concept of disability is associated with a dynamic and procedural vision [9,10,11], a dynamic process that concerns the interaction of a person, his/her health, and environmental factors. When the interaction of a person, their characteristics, and a supportive and barrier-free environment occurs, the person is enabled and supported; when this interaction occurs in an environment that is not supportive and when barriers overcome supports, the person undergoes a negative process of disabling. This vision of disability implies a universal process that could concern anyone, as anyone could be “disabled” if placed in an environment that is not adequately supportive and/or in which there are barriers and obstacles. Although this concept implies a dimensional and procedural vision, we believe it appropriate to note that sometimes, it becomes necessary to support a categorical and dichotomous vision in the field of disability, on the basis of which some people are “categorized” as having disabilities. How does one apply these concepts to the field of telemedicine and ensure that the interaction between the person and the virtual environment of telemedicine is virtuous and non-disabling? The UN Convention on the Rights of Persons with Disabilities offers possibilities for reflection, particularly by focusing on concepts/issues that are relevant in the field of e-health and telemedicine: the concepts of reasonable accommodation and Design for All. Let us begin by defining these two concepts, which are well described in article one of the aforementioned Convention [12].

The concept of reasonable accommodation refers to “all necessary and appropriate modifications and adjustments not imposing a disproportionate, undue burden, where needed, in a particular case, to ensure to persons with disabilities the participation and inclusion and exercise on an equality with all others of all human and fundamental rights”. Thus, the concept of reasonable accommodation refers to this set of personalization tools or technologies that every person with disabilities has the right to use in order to participate and to be included/to access to environments, processes, products, places, communication tools, and content. An example of reasonable accommodation is an expanded keyboard, which can facilitate the use of computers for those who have difficulty with fine motor skills that would limit their ability to use other keyboards. The concept of reasonable accommodation implies that the person can use personalization tools/strategies and elements of assistive technology to use a product, a process, or an environment. Sometimes the person uses their own tools and strategies; other times, according to article one of the UN Convention, they can use tools and strategies that are already available in the environment.

Relating this concept to the field of medicine and telemedicine, an interesting example of reasonable accommodation is the use of a braille display or the use of a screen reader to read text through speech synthesis (software that transforms digital text into audio). Both of these examples of reasonable accommodation may enable person access to digital medical reports that are provided in PDF format. In the absence of a braille bar, speech synthesis, and/or a screen reader, a person with severe visual impairment could not access such reports.

The second principle is that of Design for All, or Universal Design. This refers to an even broader vision than the previous one whereby products, environments, programs, and services are designed to be usable/enjoyed by all people, to the greatest extent possible, without the need for further adaptations or specialized planning. In the UN Convention on the Rights of Persons with Disabilities, it is explicitly stated that in the concept of Universal Design, or Design for All, products, environments, programs, and services must be designed to be used by and accessible to people with disabilities whose characteristics differ; therefore, these services must be designed to be usable by everyone. In the past, there was a different vision of design, based on the idea of an “average user”, while today, we consider the so-called “extended user”, which can involve much internal heterogeneity. If we relate this principle to the field of medicine and telemedicine, the use of the Universal Design approach implies that processes, products, environments, or information in the digital environment must be designed while considering the extreme heterogeneity of possible users and made accessible to the greatest extent possible. Article one of the UN Convention on the Rights of Persons with Disabilities also highlights that the concepts of Design for All and reasonable accommodation are not mutually exclusive; rather, they must be integrated, because Universal Design does not exclude the possibility that each individual person may require further reasonable accommodation to take advantage of these specific elements. So, relating these two concepts to the context of telemedicine and e-health, Universal Design implies the design of platforms, services, tools, environments, products, and information that can be used by people who are very different from each other, and which must therefore be designed to be accessible to and usable for each individual by taking into account these great individual differences; however, this does not exclude that each person may also require the integration of further elements of reasonable accommodation or other technologies with the previous ones.

Returning specifically to the topic of telemedicine and e-health, those who design tools, processes, products, and information in this area should consider that users can be very different from each other. Therefore, people may differ in their need for tools and assistive technologies; with respect to their sensory, perceptive, and cognitive characteristics; and with respect to their previous use of similar instruments. Additionally, healthcare professionals who use the tools and methods of telemedicine and e-health should be aware of these great individual differences and of any need for personalization and/or assistive technologies to allow every individual to benefit from these processes, tools, products, and information. Finally, users of the tools and methods of telemedicine should be given the opportunity to use these processes, tools, products, and information through the availability of assistive technology tools.

Therefore, the concepts of reasonable accommodation and Design for All are strategic in the organization of telemedicine interventions, since their accessibility is strongly linked to the reflections that have already been made in the planning phase, which should have been based on the principles of Design for All; however, it is also necessary to consider the possibility that a person with disabilities might integrate other personal assistive technologies. An equally important aspect to note is that people with disabilities can also be present among health professionals, and usability and personalization accommodations should also be afforded to health professionals themselves.

We intend to propose some reflections concerning a third area, and we will focus on keywords relating to the digital knowledge and/or skills necessary to use the tools and methods of telemedicine, as well as the infrastructures (availability of network) and the necessary devices (computers, tablets, smartphones, and other specific tools).

Therefore, considering the needs of the health professionals and users of processes, tools, products, and information, another particularly relevant concept is that of digital literacy. This refers to the set of preliminary and basic knowledge/skills necessary to use digital technologies at a basic level; these same skills and prior knowledge underlie the acquisition of more sophisticated skills. The concept of digital literacy in the context of telemedicine and e-health assumes considerable importance regarding users (patients and formal and informal caregivers), as well as healthcare operators and professionals who are not only users of the telemedicine system, but also use this telemedicine system to carry out their clinical/professional activity. The concept of digital literacy is quite interesting as it allows us to think about the “digital gap” and “digital divide”, and to make a distinction between so-called digital natives (the young regenerations who were born in a period of digital technology) and so-called digital immigrants (the previous generations for whom technology was developed at a more advanced stage of life and for whom there has been subsequent learning) [13]. This discourse is relevant not only to users of telemedicine and e-health, but also to professionals who could be digital immigrants rather than digital natives.

This distinction is particularly relevant as some people may have previous digital literacy due to being “digital natives”, while others may be “digital immigrants”, and therefore, may not yet have developed the basic skills or knowledge to use digital technologies [13]. However, both groups of people may find themselves in a position in which they must use digital technologies in a telemedicine or e-health process/service, either as healthcare professionals or as users. The topic of digital literacy therefore raises questions regarding which basic skills may already be present and which must be shared and taught to allow access to telemedicine and e-health processes/services. Other questions concern whether digital literacy is enough; therefore, basic skills and knowledge or more advanced skills are required to use telemedicine services and processes. These questions could be even more relevant when referring to healthcare professionals who must use telemedicine tools and processes and digital technologies in their clinical and professional roles. These aspects therefore highlight the importance of awareness-raising training interventions to provide the necessary basic skills and knowledge to users of telemedicine services and healthcare professionals.

These aspects are all crucial for elderly people, because it should not be assumed that they have digital literacy, and specific training programs are needed to support them in accessing to newer devices, tools, and instruments. A separate discussion should be carried out for people with disabilities, however, as they may already have previous experience of using technology in the context of the described assistive technologies; therefore, they could be more familiar with digital technologies thanks to previous use and familiarization. With the extensive use of digital technologies in the different phases of the COVID-19 pandemic, for example, people with disabilities may have had an advantage over others with less experience in the use of digital technologies and less digital literacy. Again, it should be noted that this hypothetical advantage of people with disabilities may not be generalizable, and there may not be an advantage for people with disabilities in more advanced stages of life. As such, there could be differences between people who have previously used digital technology and those who have not. From this point of view, it may also be useful to remember the two possible trajectories in the relationship between disability and aging: “disability with aging” (people who encounter disabling conditions in the most advanced stages of life due to pathologies linked to age who are unlikely to have been previously exposed to the use of technologies, assistive technology tools, or digital technologies) and “aging with disability” (which refers to people who have become aware of their disabling conditions in the initial stages of life, and are now are able to live longer due to increased life expectancy) [5,14,15,16].

Taking into consideration experience with digital technologies and digital literacy, the second trajectory may be more advantageous than the use of e-health technologies and telemedicine, precisely because these people have certain familiarity with technologies in general and digital technologies specifically.

Having made these clarifications, it is important to be able to consider the theme of accessibility together with the theme of digital literacy, and therefore, of familiarization and digital literacy. This concerns all people involved in telemedicine and who may have previously acquired familiarization and digital literacy (both users and operators), but considers that the concept of digital literacy implies basic competence which may not necessarily be sufficient for using digital tools appropriately. One could therefore ask what level of competence is necessary for patients using telemedicine tools, and what level is necessary for those who use them in a professional context such as telemedicine professionals.

For this reason, it is important not only to evaluate digital literacy, but also to understand exactly what specific level of digital competence is necessary for each of the actors involved, and how they can make adequate use of the tools, processes, and environments and avoid finding themselves in difficult conditions. A further important distinction is that even for those who are considered “digital natives”, it is important to evaluate their digital literacy and to assess their digital competence, knowledge, and ability required in the flow of specific situations; this is because sometimes, the existing skills may not be sufficient for more complex tasks which involve the use of specific areas of digital technologies. Therefore, paradoxically, even digital natives themselves may need to refine their knowledge, and it would be useful to assess their starting level in specific knowledge and skills.

Finally, we deem it useful to briefly focus on the infrastructures (availability of the network connection) and the necessary devices (computers, tablets, smartphones, and other specific tools) to enable the use of telemedicine tools and interventions. The recent COVID-19 pandemic has enabled us to reflect on this, with reference to similar areas in which the massive and sudden use of infrastructures has been necessary; we refer, in particular, to distance learning in school and university contexts and experiences of using digital platforms in the workplace, in so-called “smart working”. In both areas, critical infrastructural issues emerged with marked differences in the area and socio-economic differences both in terms of the availability of the network connection and in terms of the availability of necessary devices [2,3,4].

In this editorial, we aimed to reflect on telemedicine with reference guaranteeing equal access and usability opportunities for people with disabilities and for elderly people. We discussed certain principles and levels of analysis and intervention that may be useful in preventing the occurrence of the feared digital paradox; according to this paradox, in the absence of adequate planning and management of the variables that affect equal access potential, individuals who would benefit the most from telemedicine and telehealth are those who could be left out. We have therefore summarized some general principles that reflect the need to consider the structural and infrastructural aspects, the basic and advanced knowledge and skills necessary to use telemedicine, and the necessary adaptations in the design and use phases (particularly for people with disabilities and elderly people) while accounting for the extreme heterogeneity of possible users. We also note the importance of considering the needs and specificities of all the actors involved, particularly health professionals, as well as formal and informal caregivers.
